# Immunomagnetic separation coupled with flow cytometry for the analysis of *Legionella pneumophila* in aerosols

**DOI:** 10.1007/s00216-023-04738-z

**Published:** 2023-05-18

**Authors:** Lena Heining, Laura Welp, Achim Hugo, Martin Elsner, Michael Seidel

**Affiliations:** 1 Institute of Water Chemistry, Chair of Analytical Chemistry and Water Chemistry, School of Natural Sciences, Technical University of Munich, Lichtenbergstraße 4, 85748 Garching, Germany; 2 Institut für Energie- und Umwelttechnik e.V., Bliersheimer Straße 58-60, 47229 Duisburg, Germany

**Keywords:** Bioaerosol, *Legionella pneumophila*, Immunomagnetic separation coupled with flow cytometry, qPCR, Cultivation

## Abstract

*Legionella* *pneumophila* are pathogenic bacteria that can be found in high concentrations in artificial water systems like evaporative cooling towers, which have been the source of frequent outbreaks in recent years. Since inhaled *L. pneumophila* can lead to Legionnaires’ disease, the development of suitable sampling and rapid analysis strategies for these bacteria in aerosols is therefore of great relevance. In this work, different concentrations of viable *L. pneumophila* Sg 1 were nebulized and sampled by the cyclone sampler Coriolis® µ under defined conditions in a bioaerosol chamber. To quantify intact *Legionella* cells, the collected bioaerosols were subsequently analyzed by immunomagnetic separation coupled with flow cytometry (IMS-FCM) on the platform rqmicro.COUNT. For analytical comparison, measurements with qPCR and cultivation were performed. Limits of detection (LOD) of 2.9 × 10^3^ intact cells m^−3^ for IMS-FCM and 7.8 × 10^2^ intact cells m^−3^ for qPCR indicating a comparable sensitivity as in culture (LOD = 1.5 × 10^3^ culturable cells m^−3^). Over a working range of 10^3^ − 10^6^ cells mL^−1^, the analysis of nebulized and collected aerosol samples with IMS-FCM and qPCR provides higher recovery rates and more consistent results than by cultivation. Overall, IMS-FCM is a suitable culture-independent method for quantification of *L. pneumophila* in bioaerosols and is promising for field application due to its simplicity in sample preparation.

**Graphical abstract:**

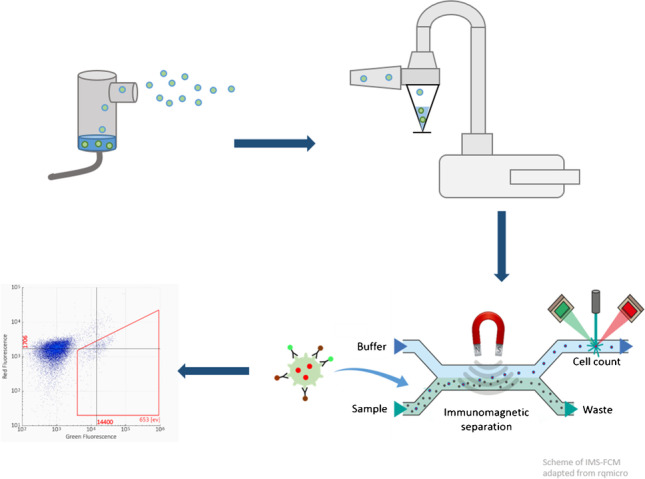

**Supplementary Information:**

The online version contains supplementary material available at 10.1007/s00216-023-04738-z.

## Introduction

Over recent years, there have been repeated news about outbreaks of *Legionella* resulting from the release of bioaerosols from evaporative cooling systems [1–3]. To establish rapid quantification of pathogens in aerosols by culture-independent methods, which are based on immunoassays or molecular biological methods, it must be proven that these approaches achieve similar results as by culture. Additionally, sampling strategies that are compatible with such rapid analysis need to be verified. This raises the need to explore ways of studying viable pathogens in bioaerosols in the laboratory without the risk of exposure. Nebulizing and bioaerosol sampling must be performed in bioaerosol chambers in laboratories of class 2 or higher [4]. In this work, such a protocol was implemented in a bioaerosol chamber [5] for the first time with active *Legionella*. These bacteria occur in more than 50 species, the most common pathogenic one being *Legionella* *pneumophila* [6]. The term *Legionella* species (*Legionella* spp.) includes all species. The specific species *L. pneumophila*, in turn, can be divided in serogroups (Sg), which are differentiated by the structure of their lipopolysaccharides (LPS), a component on the outside of their membranes [7]. *L. pneumophila* Sg 1 is the most frequent cause of infection, accounting for over 70% of cases, and it is the most common causative agent for the disease legionellosis [8, 9]. This includes Legionnaires’ disease, an infection similar to pneumonia, and Pontiac fever, a milder form that resembles a cold and often goes unnoticed [10]. A common exposure way is the inhalation of contaminated bioaerosols within a size range of 1–5 µm, as those airborne particles reach the alveoli area in the lung where they can cause an infection [11]. A very frequent polluter of *Legionella* in bioaerosols are evaporative cooling systems. In such systems, process water is cooled by heat exchange between water and air. To increase the cooling effect, the water is nebulized to generate small droplets. If the process water is contaminated with *Legionella*, the chance is given that they are transported in droplets to the outside of the tower up to several kilometers away from the source where they represent a health risk when inhaled [12]. Since in case of an outbreak fast laboratory results are necessary to identify the source of a contamination, rapid detection methods are needed.

The analysis of *Legionella* by cultivation is still the gold standard, even though cultivation comes with many disadvantages. First, the long analysis time of up to 10 days bears the risk of possible outbreaks of *Legionella* before results become available. Second, the presence of other microflora can overgrow *Legionella* colonies, so that a quantification may prove difficult. Third, *Legionella* can enter a viable-but-not-culturable (VBNC) state, making them undetectable through cultivation, which leads to an underestimation of the concentration [13–16]. Dietersdorfer et al. [17] showed that VBNC *Legionella pneumophila* Sg 1 are still virulent in human macrophages, albeit with reduced efficiency. This is of particular importance in the case of aerosols, as the collection process for analysis can cause bacteria to enter stress situations and switch to the metabolically inactive status. Although they are still pathogenic, they will not be detected by cultivation.

For these reasons, the establishment of culture-independent analytical methods for bioaerosols is highly recommended. A molecular biological technique for the detection of *L. pneumophila* is the quantitative polymerase chain reaction (qPCR). In addition to high sensitivity and specificity [18–21], it has a low limit of detection (LOD) of, for example, 1.6 × 10^2^ genomic units (GU) L^−1^ [20] or 80 GU L^−1^ [21]. With the ability to measure 96 samples at once and to determine the distribution between living and dead cells, there are many advantages of this bioanalytical method. On the other hand, a DNA extraction has to be performed beforehand, which requires skilled personnel to minimize loss of DNA. In environmental samples, inhibiting compounds also represent a source of error [13, 15, 22, 23]. Despite these drawbacks, qPCR is a promising culture-independent method for the detection of *L. pneumophila* in water and aerosol as already demonstrated in past research [19, 24, 25]. A distribution between intact and damaged cells becomes also possible with qPCR when adding a dye that binds covalently to the DNA of cells with damaged cell membranes and inhibits the amplification [26]. Propidium monoazide (PMA) and ethidium monoazide (EMA) are examples of such dyes [25–27].

The immuno-analytical platform rqmicro.COUNT, which relies on a combination of antibody-based immunomagnetic separation (IMS) and flow cytometry (FCM) in a microfluidic plastic cartridge, is a promising new measurement system for the rapid detection of *L. pneumophila*. The general advantage over other flow cytometry systems is that no washing steps of the fluidic system are needed, which reduces maintenance and unwanted carry-over. Furthermore, the combination of IMS and FCM enables measurements in complex matrices which is often a big challenge of FCM [28, 29]. It was shown before [30–32], that in principle IMS coupled with FCM is suitable for the analysis of *L. pneumophila* in different water matrices, but the approach has not yet been investigated with aerosols. Here, magnetic particles are coupled to a panel of monoclonal anti-*L.* *pneumophila* Sg 1 antibodies that enable the separation of bacteria cells from other particles of the matrix. The quantification via FCM takes place through the addition of green fluorochromes, also coupled to anti-*L. pneumophila* Sg 1 antibodies, and in the following referred to as staining dye [33]. Advantages of this method are the low analysis time of 2 h and the absence of elaborate sample pre-treatment. Through the further addition of the red dye propidium iodide (PI), which only enters cells with damaged cell membranes, a distribution between intact (viable) and damaged (dead) cells becomes possible [26].

So far, the analysis of process water is preferred over that of the emitted air since an easier sampling can be applied. Nevertheless, the analysis of aerosols can have benefits, for example for testing drift eliminators efficiencies. In addition, it is not fully investigated whether other sources, like biofilm in the cooling tower, can lead to an emission of *Legionella*. To enable direct sampling of bioaerosols, a suitable aerosol sampler with sufficient physical and biological sampling efficiency is required. Hereby, the physical sampling efficiency is the recovery of the particles in the collected aerosol, whereas the biological sampling efficiency states additionally the survival of bacteria during the collection process [34]. Because of difficulties in the decontamination of particle counters, it is challenging to measure the total amount of particles for pathogens. Therefore, in our work, the sampling efficiency of total and viable *Legionella* is determined by combining the sampler with the respective detection method. By nebulizing a defined bacteria concentration, a calibration of the measurement system can be achieved.

There are various kinds of aerosol samplers available which show different sampling efficiencies. With cyclone sampling, in this case the Coriolis® µ, the cells are captured in a liquid, which improves the viability of bacteria through less drought stress [35]. When entering the sampler, the air flows in a spiral pattern causing a vortex in which particles larger than the cut-off diameter accumulate on the walls due to inertia and centrifugal forces. The air then leaves the sampler through an outlet on the top [36]. These kinds of samplers are less prone to re-entrainment of particles than other sampling techniques like impingement or impaction [37, 38] and are suitable to collect particles above a size of 0.5 µm according to the manufacturer. With an airflow of 100–300 L min^−1^, it shows a high sampling volume compared to other samplers. Previous experiments with inactivated *L. pneumophila* in aerosols indicated a sampling efficiency of 42% for the Coriolis® µ [39], but so far, no studies have been conducted with living *Legionella.*

In this study, *L. pneumophila* Sg 1 of defined concentrations was nebulized with specified droplet sizes in a bioaerosol chamber and subsequently collected with the cyclone sampler Coriolis® µ. For the first time, to our knowledge, the IMS-FCM method was applied to analyze *L. pneumophila* in aerosols. This measuring system was then compared to cultivation and qPCR to evaluate their suitability for analysis of *L. pneumophila* in aerosols. Total *Legionella* count (TLC) as well as intact *Legionella* count (ILC) were compared to derive the physical and biological sampling efficiency of the Coriolis® µ sampler depending on the cell concentration and the used analytical detection method.

## Material and methods

### Bacteria cryo standard

Bacteria solutions were obtained from a *L. pneumophila* Sg 1 Subtype Bellingham cryo standard (produced from strain DMSZ 25214, see Supplementary Information) with a TLC of 4.82 × 10^7^ cells mL^−1^ and an ILC of 4.77 × 10^7^ cells mL^−1^. Cryo stocks are a 1:1 mixture of bacteria suspended in Evian water (purchased from local store) and cryo buffer (122 g L^−1^ K_2_HPO_4_, 14 g L^−1^ KH_2_PO_4_, 85 g L^−1^ NaCl, 20 g L^−1^ BSA, and 120 g L^−1^ Dextran 40 in deionized water, all chemicals from Sigma-Aldrich, St. Louis, USA). The produced cryo stocks were stored at  − 80 °C until further use. Through dilution in Ringer’s solution (B.Braun, Melsungen, Germany), the intended concentrations of bacteria solutions were achieved.

### Preparation of aerosol samples

For the aerosolization, four different concentrations between 10^3^ and 10^6^ cells mL^−1^, relating to TLC, and sterile Ringer’s solution as a blank (0 cells mL^−1^) were nebulized and sampled. The ILC is slightly less (99% ILC) than the TLC. Therefore, concentrations in the range of 9.87 × 10^1^ cells mL^−1^ to 9.92 × 10^5^ cells mL^−1^, relating to ILC, were achieved. Five milliliters of the bacterial solutions was put in the nebulizer vessel. For each concentration, three bacterial solutions and one blank were nebulized. All nebulizer vessels were weighed before and after the nebulizing process to obtain the amount of generated aerosol. Collection vessels of the Coriolis® µ sampler were filled with 10 mL sterile Ringer’s solution as collection liquid. The vessels were weighed before the filling and after the sampling to determine the remaining amount of sample. Because of liquid loss due to evaporation, the vessels were filled up to 10 mL with sterile Ringer’s solution afterwards.

### Collection of aerosols

Aerosol generation and collection took place in a bioaerosol chamber in a Bio2 laboratory. The modified glove box has HEPA-filters on openings for incoming and outgoing air so a constant air flow through the chamber can be realized. It is operated at negative pressure to avoid any safety risks while working with pathogen aerosols. The chamber is described in detail elsewhere [5]. Sampling was done with the cyclone sampler Coriolis® µ (Bertin, Montigny-le-Bretonneux, France), while nebulizing was performed with a PARI LC PLUS® Nebulizer (Pari GmbH, Starnberg, Germany) and a PARI BOY® Compressor (Pari GmbH, Starnberg, Germany). Nebulizer and sampler were started at the same time and aerosols were collected for 10 min with a flow rate of 300 L min^−1^. Afterwards, collection vessels were removed from the chamber through a sluice for further analyzation.

### Measurements with IMS-FCM

For measurements with IMS-FCM on rqmicro.COUNT, the samples needed to be contained in a defined medium (10 mM phosphate buffer with pH 7.4, 150 mM NaCl/KCl, 1% BSA, 0.05% Tween-20) for proper interaction with the antibodies of magnetic particles and staining dye. Therefore, a solution containing 100 mM phosphate (80 mM Na_2_HPO_4_, 20 mM KH_2_PO_4_), 10% BSA, and 0.5% Tween-20 was prepared in deionized water (all chemicals from Sigma-Aldrich, St. Louis, USA) and subsequently diluted 1:10 in the sample (therefore results need to be multiplied with a factor of 1.11). The required chloride concentration was already covered by Ringer’s solution. Ten microliters of magnetic particles Sg 1 (rqmicro, Schlieren, Switzerland) and 10 µL staining dye Sg 1 (rqmicro, Schlieren, Switzerland) were added to 200 µL of the prepared samples followed by an incubation for 1 h at RT on an overhead shaker (rqmicro, Schlieren, Switzerland). After incubation, 800 µL of buffer 1 (rqmicro, Schlieren, Switzerland) was added to the samples. One milliliter thereof was then transferred into the cartridge (rqmicro, Schlieren, Switzerland). For determination of TLC, 2 mL of 1 × PBS (137 mM NaCl, 2.7 mM KCl, 8 mM Na_2_HPO_4_, 2 mM K_2_PO_4_) and 0.05% Tween-20 were added to the buffer wells; for ILC measurements, 2 mL of PI containing buffer 2 (rqmicro, Schlieren, Switzerland). Magnetic particles, staining dye, buffer 1, and buffer 2 were part of the rqmicro L.p. SG1 DETECT Kit (31010) (rqmicro, Schlieren, Switzerland). Measurements were performed on the device rqmicro.COUNT (rqmicro, Schlieren, Switzerland) that combines immunomagnetic separation with flow cytometry. With one cartridge, four samples could be measured simultaneously within 49 min. All samples were measured in triplicate.

### DNA extraction

DNA extraction was performed with a foodproof® StarPrep Two Kit (Biotecon, Potsdam, Germany). Therefore, 700 µL of the sample was used without further treatment except for determination of living cells, where 300 µL of D-Reagent (Biotecon, Potsdam, Germany) was added as well. All further steps were conducted according to the manual of the kit. Prepared DNA extracts were stored at  − 20 °C until use.

### Measurements with qPCR

qPCR measurements were performed with a microproof® *Legionella* Quantification LyoKit (Biotecon, Potsdam, Germany) according to ISO/TS 12869:2019, where 25 µL of DNA extract was added to the Quantification Kit according to the manual. Afterwards, qPCR was conducted on a qPCR Tower^3^ G (Analytik Jena, Germany). A negative control (PCR-H_2_O) and two positive controls (standards A and D from the Quantification Kit) were added to all measurement runs to check that the system worked properly. The measuring program was run with thermal cycling conditions stated in the manual. In one run, *L. pneumophila*, *L. pneumophila* Sg 1, and *Legionella* spp. were measured simultaneously. All DNA extracts were measured in triplicate.

### Cultivation

0.1 mL, 0.3 mL, and 0.5 mL of each sample were plated on BCYE agar plates (Xebios Diagnostics, Düsseldorf, Germany) with different dilutions. The plates were incubated at 37 °C for 10 days in a CO_2_ incubator (Binder, Tuttlingen, Germany). Colonies were counted after 5, 7, and 10 days.

### Data evaluation

For aerosol measurements, the aerosol factor had to be considered. It refers to the volume of nebulized bacteria solution relative to the end volume in the collection vessel, which is 10 mL in our experiments. This factor serves to convert measured counts from collection vessels to the number of *L. pneumophila* in aerosols.

1 $$Aerosol\;factor=\frac{End\;volume\;in\;collection\;vessel}{Volume\;of\;nebulized\;bacteria\;solution}$$

Recoveries before (Recovery_assay_) and after (Recovery_aerosol_) aerosolization were calculated as follows:

2 $${Recovery}_{assay}=\frac{{Measured\;concentration}_{before}}{Applied\;concentration}\times100\%$$

3 $${Recovery}_{aerosol}=\frac{{Measured\;concentration}_{aerosol}}{{Measured\;concentration}_{before}}\times100\%$$

The LOD for aerosols (LOD_aerosol_) was determined with the following equation as stated elsewhere [39].

4 $${LOD}_{aerosol}=\frac{{LOD}_{method}\times {V}_{end}}{Q\times t}\frac{1}{\eta }$$

where LOD_method_ is the LOD of the respective analytical method, *V*_end_ is the end volume in collection vessel, *Q* is the flow rate of the sampler, *t* is the sampling time, and *η* is the sampling efficiency. Sampling efficiency equals recovery_aerosol_ (see Table 1).

## Results and discussion

### Droplet spectrum

As a result of the characterization of generated aerosols by PARI LC PLUS® nebulizer, Fig. S1 shows the cumulative mass distribution. Fifty percent of the mass fraction falls within a droplet size range above and below a mass mean diameter (MMD) of 6.3 µm, respectively, whereas 80% of the mass falls in the range between 2.3 and 12 µm. As it is stated elsewhere [40], the generated droplet sizes in our experiments are in the right range to not only carry bacteria cells but also to reach the thoracic region as well. Therefore, they are suitable to simulate the droplets in the environment that can cause an infection. In addition, according to Carvalho et al. [41], the Coriolis® µ sampler shows physical sampling efficiencies between 41 and 92% for particles with a diameter of 2.4 to 10 µm. While these values are not directly comparable to our results due to differences in experimental conditions, they provide a rough estimate of what to expect.

### Experimental setup

**Fig. 1:**
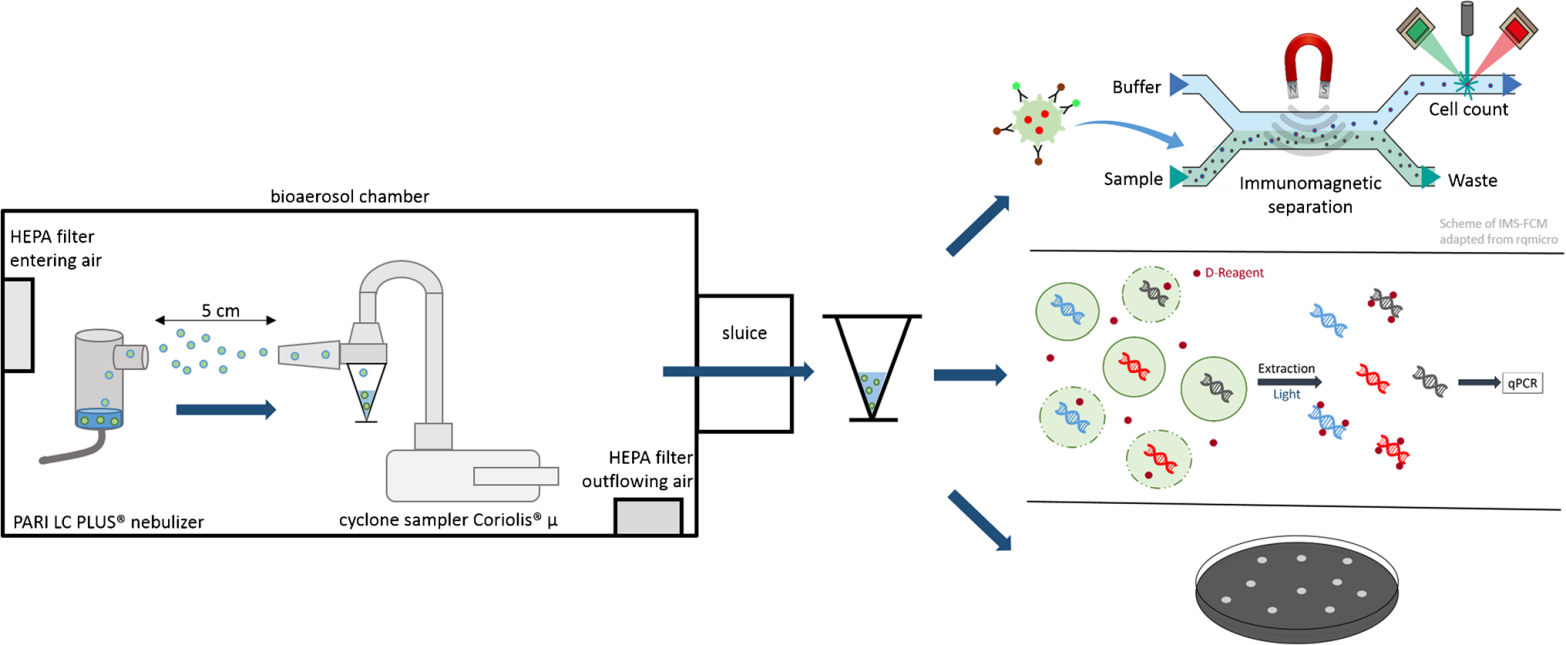
Experimental setup for experiments with aerosols. Bioaerosols were generated with a PARI LC PLUS® nebulizer and collected with the cyclone sampler Coriolis® µ in a bioaerosol chamber. Nebulizer and sampler were placed within a distance of 5 cm. After finishing the sampling process, the collection vessels were taken out of the chamber through a sluice. By adding magnetic particles and a staining dye, the IMS procedure and FCM measurements were performed successively on the measuring device. In addition, a DNA extraction followed by qPCR and analysis by cultivation were conducted

To characterize any bioanalytical method for pathogens in bioaerosols, nebulizing and aerosol sampling has to be performed in a bioaerosol chamber as shown in Fig. 1. Throughout the experimental setup, it was possible to perform experiments with viable *L. pneumophila* Sg 1 in bioaerosols without the risk of exposure. Cyclone sampling is a favorable method for many applications because the bacteria cells are transferred from air into an aqueous medium from which sample detection can directly be taken.

#### IMS-FCM

**Fig. 2:**
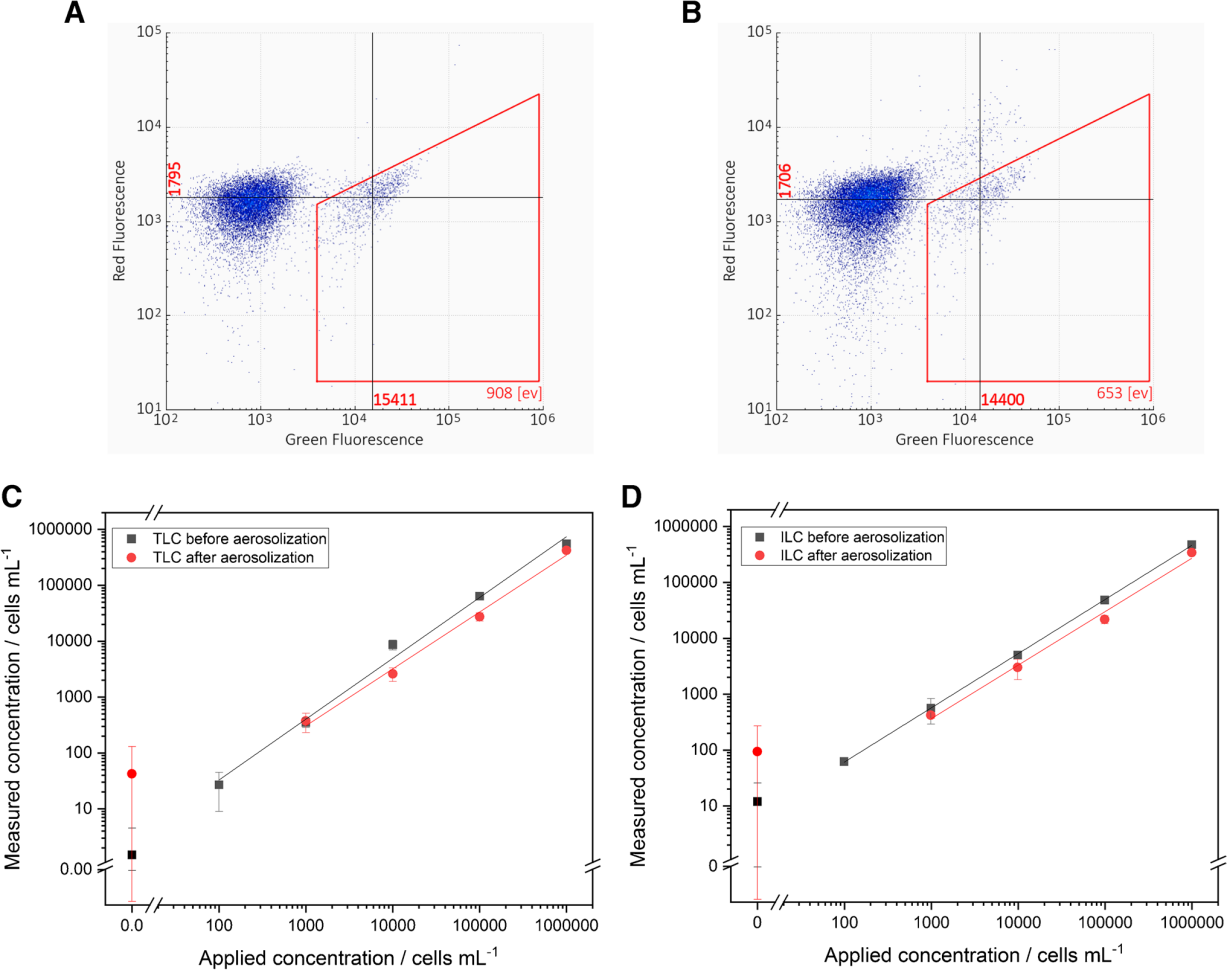
**A**, **B** Received dot plots from the measurements of green and red fluorescence with IMS-FCM of nebulized 10^5^ cells mL^−1^. **A**, TLC; **B**, ILC, with dead cells shifted to red fluorescence. **C**, **D** Correlation between applied and measured concentration in cells mL^−1^ of *Legionella* *pneumophila* Sg 1 for IMS-FCM. Measuring points (number of measurements (m) = 4 for aerosol; m = 5 before aerosolization) before (spiked concentration in nebulizer vessel) and after aerosolization (found concentration in Coriolis® µ vessel) were added. **C**, TLC and **D**, ILC. Error bars represent replicate measurements (number of replicates (*n*) = 3)

For data evaluation, the IMS-FCM analysis platform rqmicro.COUNT generates dot plots by plotting green against red fluorescence. A default gate, which was set by the manufacturer for drinking water and was adjusted for aerosol samples, was used for counting of events. The adjustment was necessary because a different matrix than drinking water was used, which can lead to a slight shift of events. To prevent events from the background in the gate, it was manually adjusted directly on the device. Blank measurements were used to distinguish between events of the background and the cells. In Fig. 2A, the dot plot of the TLC measurement for 10^5^ cells mL^−1^ nebulized *L. pneumophila* Sg 1 is shown. On the left outside the gate, the background noise of the device is visible, while events in the gate represent stained bacteria cells. As here only the green staining dye was used, intact and damaged cells are both measured. Accordingly, the total count of *L. pneumophila* Sg 1 results in 3.3 × 10^4^ cells mL^−1^ after considering dilution factors. In comparison, Fig. 2B shows the result after adding PI which intercalates with double-stranded DNA of damaged cells. This means damaged cells are shifted in the direction of red fluorescence and are now outside the gate. The events remaining in the gate represent bacteria with intact cell membranes which include active and VBNC *L. pneumophila* Sg 1. A concentration of 2.4 × 10^4^ ILC cells mL^−1^ was determined for this sample.

For TLC as well as for ILC, recovery_assay_ without aerosolization (Eq. 2) and recovery_aerosol_ (Eq. 3) are responsible for loss in cell concentration compared to the applied bacteria concentration. For recovery_assay_, 53.7 ± 23.8% for TLC and 52.9 ± 6.2% for ILC were calculated. Both recoveries show similar results for ILC as well as for TLC, so the system is suitable for both kinds of measurements. For TLC, a 17.6% higher standard deviation is seen, which can be justified by the different techniques. During TLC measurements, antibodies of staining dye and magnetic particles bind to all cells with LPS structures of *L. pneumophila* Sg 1, even those with damaged cell membranes. Damaged cells have the disadvantage of coagulation effects, which can interfere with the measurements. For ILC measurements, only intact cells are considered and coagulation effects are reduced.

Since the results received with IMS-FCM are given as cells mL^−1^ for TLC and ILC, respectively, they can directly be correlated to those of the applied concentrations in the nebulizer. The plotting of applied against measured concentration for TLC before aerosolization (Fig. 2C) demonstrates a linear correlation (Pearson *r* (*ρ*) = 0.996, number of measurements m = 5, for linear regressions, see Table S1). After aerosolization, there is still an identical linear correlation between applied cells and sampled cells (*ρ* = 0.997, m = 4). By looking at the measurements of ILC (Fig. 2D), similar results compared to TLC can be recognized. A linear correlation before (*ρ* = 0.999, m = 5) and after (*ρ* = 0.996, m = 4) aerosolization is given.

Blank measurements with nebulized Ringer’s solution were also added to the graph. When comparing the results of these measurements before and after the aerosolization, an increase of 41.0 cells mL^−1^ for TLC and of 82.9 cells mL^−1^ for ILC is visible. This can be explained by possible carry-over during the collection process with the sampler in the aerosol chamber, where the possibility of remaining bacteria in the air or the sampler is given.

In addition, the LODs for ILC and TLC were calculated. Here, it can be distributed between LOD of the detection method (LOD_method_) and the LOD with aerosols (LOD_aerosol_). LOD_method_ was calculated by adding three times the standard deviation of blank measurements to the mean value of blank measurements in aerosols. This results in 2.4 × 10^2^ cells mL^−1^ for applied TLC and 5.5 × 10^2^ cells mL^−1^ for applied ILC. With these results, the LOD_aerosol_ could be calculated by using Eq. (4), with *V*_end_ = 10 mL; *Q* = 0.3 m^3^ min^−1^; *t* = 10 min; and *η*_TLC_ = 0.64, *η*_ILC_ = 0.63. This resulted in LOD_aerosol__, __TLC_ = 1.3 × 10^3^ cells m^−3^ and LOD_aerosol__, __ILC_ = 2.9 × 10^3^ cells m^−3^.

All these results lead to the assumption that this method coupled with aerosol sampling is suitable to gain consistent results for *Legionella pneumophila* Sg 1 in aerosols.

### qPCR

**Fig. 3:**
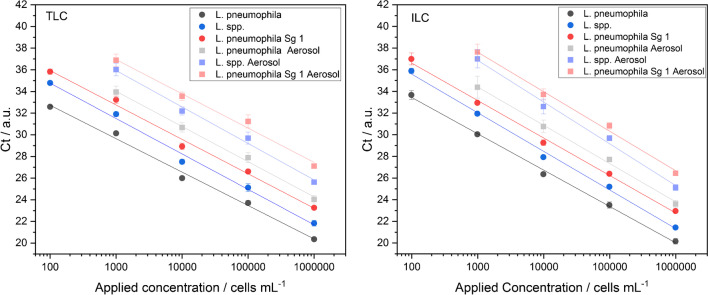
Correlation curves (m = 5 (before aerosolization); m = 4 (aerosol)) between applied concentrations and Ct values measured by qPCR. Results are given for three different genes before (spiked concentration in nebulizer vessel; brighter colors) and after (found concentration in Coriolis® µ vessel; lighter colors) aerosolization. Results for TLC on the left, for ILC on the right. Error bars represent replicate measurements (*n* = 3)

With the used qPCR kit, genes specific to L. *pneumophila*, *L. pneumophila* Sg 1, and *Legionella* spp., referred to as target genes, can be determined simultaneously. Figure 3 shows the measured Ct values before as well as after aerosolization for the respective concentrations. Because *L. pneumophila* Sg 1 was used for the experiments, positive results for all three target genes are expected.

Plotting of applied concentrations before aerosolization against measured Ct values demonstrates a linear correlation for TLC as well as for ILC in the range of 10^2^–10^6^ cells mL^−1^ for all three target genes (all *ρ* ≥ 0.997, m = 5). After the nebulizing and sampling process, no decline in the correlations can be identified (*ρ* ≥ 0.997, m = 4). This indicates that the extraction process works equally consistent even with different concentrations for *Legionella* in aerosols and suggests that the measurement system is suitable for these kinds of measurements.

In addition, blank measurements with nebulized Ringer’s solution were performed before and after aerosolization but no Ct value could be obtained. Since with IMS-FCM an increase in blank values after aerosolization was found, this increase would be expected to occur as well with qPCR but was not confirmed. It is likely that the concentrations in the blank may be too low to detect with qPCR. For determination of concentrations of *Legionella* in aerosols, measurements before aerosolization were used as a calibration. The respective linear equations are summarized in Table S2.

For calculation of LOD_aerosol_, Eq. (4) is applied. Since no value for the blank measurements could be determined, 100 cells mL^−1^ (TLC) and 98.7 cells mL^−1^ (ILC) were used as the lower limit of the working range of the method because it was the lowest measured concentration before aerosolization in our experiments that shows a positive signal and is in the linear range. This value is comparable to the LOD of 65 GU mL^−1^ specified by the manufacturer. The comparability of our results with LODs from literature, as stated before, indicates a suitable DNA extraction with minimal loss. The first step in the extraction process is centrifugation to form a pellet. After adding lysis buffer and performing the extraction, the whole amount of liquid is removed as DNA extract for further measurements. This presumably leads to a high yield of DNA at the end. With *V*_end_ = 10 mL; *Q* = 0.3 m^3^ min^−1^; *t* = 10 min; *η*_TLC_ = 0.35; and *η*_ILC_ = 0.42, LOD_aerosol__,_ _TLC_ = 9.5 × 10^2^ cells m^−3^ and LOD_aerosol, ILC_ = 7.8 × 10^2^ cells m^−3^ were calculated.

### Cultivation

**Fig. 4:**
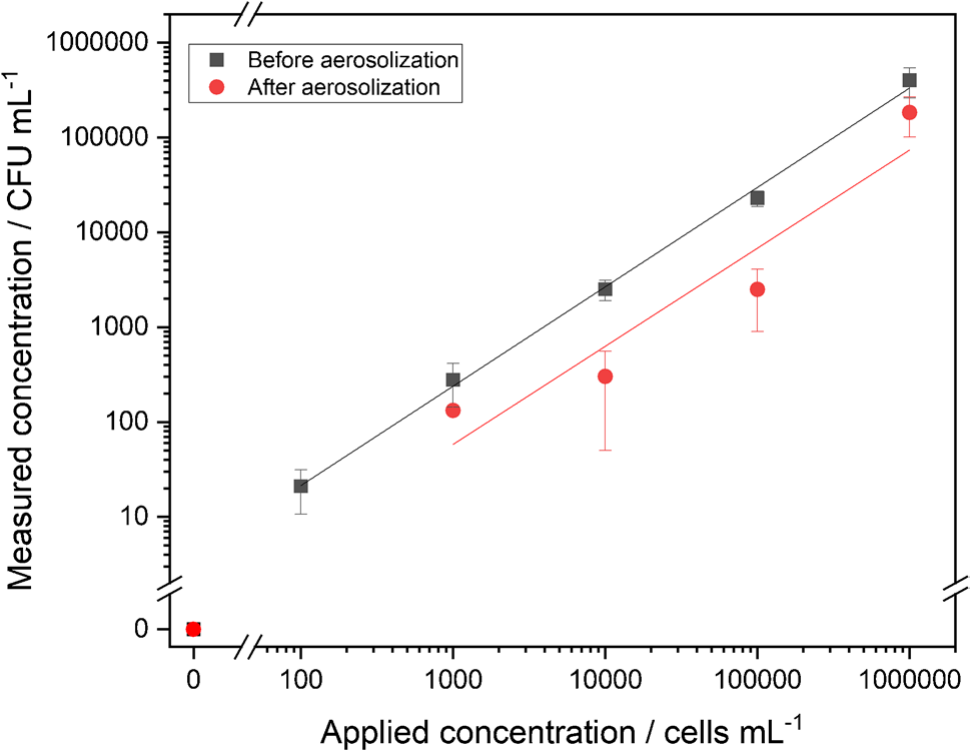
Correlation curves between applied and measured concentrations before (spiked concentration in nebulizer vessel, m = 5) as well as after aerosolization (found concentration in Coriolis® µ vessel, m = 4) quantified by cultivation. Error bars represent replicate measurements in triplicate (*n* = 3)

For cultivation, the nebulized sample concentrations were given in cells mL^−1^, but results were obtained in CFU mL^−1^. Comparing applied concentrations to the number of colonies on the plates (Fig. 4), the decrease in found concentration showed that even before aerosolization only 27.5 ± 7.5% of the cells formed a colony.

However, we can state that there is a linear correlation between applied cells and measured concentration before aerosolization (*ρ* = 0.999, m = 5). By comparing this with results after aerosolization, it can be recognized that there is a reduced linear correlation (*ρ* = 0.950, m = 4) and recovery_aerosol_ is less (30.9 ± 18.0%), compared to IMS-FCM and qPCR. It should also be mentioned that some results with aerosols show an error bar’s overlap through high standard deviations. This can be reasoned with the presence of bacteria in the VBNC state which is induced by the nebulizing and sampling procedure of the aerosols. It indicates that the percentage of *L. pneumophila* in this state is not always the same, which results in high standard deviations. This leads to the conclusion that the Coriolis® µ sampler may impact the physiological state of cells and is also a confirmation for the need to establish culture-independent methods.

LOD_method_ was calculated considering the recommendation of the German Federal Environmental Agency [42], which states that results are significant when three or more colonies can be counted on the growth medium. In our experiments, we used 0.1 mL as the minimal sampling volume so LOD_method_ would be 30 CFU mL^−1^, which equals 1.4 × 10^2^ cells mL^−1^. With *V*_end_ = 10 mL; *Q* = 0.3 m^3^ min^−1^, *t* = 10 min, and *η* = 0.31, a LOD_aerosol_ = 1.5 × 10^3^ cells m^−3^ was calculated.

### Comparison of applied methods

When comparing the recoveries in the aerosol, it has to be noted that with ILC measurements for IMS-FCM and qPCR, intact cells (active and VBNC) are measured, whereas for cultivation, only culturable cells are determined. With IMS-FCM, only *L. pneumophila* Sg 1 is detectable through antibodies, while with the applied qPCR kit, three different target genes can be analyzed. With cultivation, it cannot be differentiated between species or serogroups, so only statements about the number of *Legionella* spp. can be made.

The recoveries of bacteria in aerosols for all three methods are summarized in Table 1. They directly demonstrate the sampling efficiencies of the Coriolis® µ in combination with different analytical methods. With the ratio of ILC to TLC, the biological sampling efficiency can be determined as well.

**Table 1:** Comparison of recovery_aerosol_ (*n* = 4) and LOD_aerosol_ for all three applied analytical methods. Results by cultivation only represent culturable cells and *Legionella* spp

	LOD_aerosol_/cells m^−3^	Recovery_aerosol_ TLC/%	Recovery_aerosol_ ILC/%
IMS-FCM			
* L. pneumophila* Sg 1	1.3 × 10^3^ (TLC) 2.9 × 10^3^ (ILC)	63.7 ± 34.1	63.0 ± 13.5
qPCR			
* L. pneumophila*	9.5 × 10^2^ (TLC) 7.8 × 10^2^ (ILC)	35.8 ± 12.1	44.9 ± 14.7
* L. pneumophila* Sg 1		35.1 ± 11.3	42.2 ± 16.1
* Legionella *spp*.*		35.3 ± 11.9	40.7 ± 15.0
Cultivation			
* Legionella* spp*.*	1.5 × 10^3^	-	30.9 ± 18.0

For IMS-FCM, 63.7 ± 34.1% of TLC and 63.0 ± 13.5% of ILC could be found after the sampling. For the combination of IMS-FCM and Coriolis® µ sampler, this shows a higher medial sampling efficiency, but also higher standard deviation compared to 42 ± 9% found in Langer et al. [39]. There, inactivated bacteria were sampled in the same way but analyzed by microarray. Other samplers, like the All-Glass Impinger 30 (AGI-30) or the Andersen cascade impactor, showed recoveries between 38–77% [39, 43] and 36–71% [44], respectively. Differences compared to the Coriolis® µ can occur through different physical sampling principles and the used analytical method. In addition, the biological sampling efficiency of the Coriolis® µ is 99%, which means that the forces during the sampling procedure are not destroying the cells, just have influence on the physiological state as the results by cultivation showed.

By looking at the results of qPCR, the three target genes show very similar results for TLC with a recovery_aerosol_ of 35.4 ± 0.4%, whereas for ILC, a recovery_aerosol_ of 42.4 ± 2.3% was obtained. This would lead to the conclusion that the mean biological sampling efficiency of the sampler would be 120.2 ± 5.0%. Normally, we would expect a decrease in survival due to strong forces in the aerosol sampler, so it is likely that the increase in survival has its source in the extraction or measurement process. The measurements of the DNA extracts for TLC and ILC were performed on different days and with the use of the aerosol factor. Even small differences in Ct values add up in the calibration curve as well as in the aerosol samples and lead to an increase of concentrations at the end. Another explanation can be the use of the D-reagent for ILC measurements that may have an influence on the higher recoveries for ILC measurements.

When comparing the results of IMS-FCM and qPCR, differences between these two methods can be seen. Since measurements of both methods were performed with the same samples, the sampling efficiency of the Coriolis® µ sampler would be expected to be identical, still there are differences between the recoveries. This can be explained either through the sampling or measurement process. For the first one that would indicate that bacteria are changed during the collection in a way that they behave different at the extraction process. During the first centrifugation step in the extraction, bacteria build a pellet on the ground of the vial. Free DNA remains in the supernatant and is therefore removed with it. It was shown before [45, 46] that centrifugation can have an influence on the integrity of cell membranes. In our experiments, it is possible that cell membranes are weakened through the forces in the sampler. Even small forces during centrifugation can now lead to a rupture of weakened cells and to a release of free DNA. Since centrifugation of the sample is not necessary with IMS-FCM, this would explain the higher recoveries with this method.

With 30.9 ± 18.0%, the lowest recovery_aerosol_ could be found with cultivation, which was expected because of the VBNC state. This confirms, as stated before in previous studies [19–21], that there is an underestimation of bacteria concentrations through cultivation. Another disadvantage of this method is the long analysis time of 10 days, whereas with IMS-FCM, a result is obtained within 2 h and with qPCR (including extraction) within 4 h. It is often stated that cultivation has a high sensitivity because even low numbers of colonies can be analyzed. However, by our results, it was demonstrated that not every cell forms a colony. Therefore, only taking colonies into account indicates an underestimation of the real number of bacteria cells. When stating the results in cells mL^−1^, LOD_aerosol_ of cultivation rises above that of qPCR. Comparing this with received LOD by IMS-FCM, values in the same range as by cultivation can be seen. In combination with a better recovery and a lower measurement time, this speaks for the practicality of the established IMS-FCM method. qPCR still shows the lowest LOD but is more laborious due to the needed DNA extraction beforehand.

Previous studies addressed the analysis of real samples of emitted air from water-bearing systems. Ishimatsu et al [47] detected 90 CFU m^−3^ around cooling towers, whereas Mathieu et al. [48] stated over 10^3^ cells m^−3^ during an outbreak in France. Blatny et al [49] found 3.3 × 10^3^ CFU m^-^^3^ at a biological treatment plant. But as the given data are mostly not stated in cells m^−3^, it is difficult to compare it to our results. Nevertheless, our analytical methods are promising to detect concentrations that occur in the environment of evaporative cooling systems.

## Conclusion

We were able to show that IMS-FCM is suitable for the rapid quantification of viable and dead *L. pneumophila* Sg 1 cells in bioaerosols with a prior aerosol sampling by the Coriolis® µ. The consistency of the results across all concentrations was demonstrated along with a high biological sampling efficiency of 99%. Additionally, we showed that this culture-independent method provides a wider range of information, such as distribution between intact and damaged cells or a defined serotype. Because of several disadvantages of cultivation regarding detection time and underestimation in aerosols due to bacteria in the VBNC state, more research about culture-independent methods is needed. In comparison to qPCR, no elaborate sample preparation is required, and results are obtained in a shorter time. Furthermore, the results with IMS-FCM showed higher recoveries for TLC and ILC.

We have shown that IMS-FCM is a simple and rapid method that is promising for field measurements to quantify emission of *L. pneumophila* from evaporative cooling or other nebulizing water systems. With this, emission measurements of *L. pneumophila* could be performed more frequently to improve Legionnaires’ disease risk assessment.

The entire experimental setup is also promising to be adapted to investigate different types of bioaerosols. This can be useful for conducting studies on the bioanalytical characterization of cultivation-independent methods with viable pathogenic bacteria or active viruses.

## Supplementary Information

Below is the link to the electronic supplementary material.

Supplementary file1 (DOCX 51 KB)
