# Supplementary material for: Immunomagnetic separation coupled with flow cytometry for the analysis of Legionella pneumophila in aerosols
Source: Anal Bioanal Chem. 2023 May 18;415(21):5139–49. doi: 10.1007/s00216-023-04738-z (PMC10404198; doi:10.1007/s00216-023-04738-z)
Supplement: Supplementary file 1 — Supplementary file1 (DOCX 51 KB) [file 216_2023_4738_MOESM1_ESM.docx]

**Supplemented information**

**Immunomagnetic separation coupled with flow cytometry for the analysis of *Legionella pneumophila* in aerosols**

Lena Heining^1^, Laura Welp^2^, Achim Hugo^2^, Martin Elsner^1^, Michael Seidel^1^*

^1^Institute of Water Chemistry, Chair of Analytical Chemistry and Water Chemistry, Technical University of Munich, Lichtenbergstraße 4, 85748 Garching, Germany

^2^Institut für Energie- und Umwelttechnik e.V., Bliersheimer Straße 58-60, 47229 Duisburg, Germany

*Corresponding author: michael.seidel@mytum.de

MATERIAL AND METHODS

**Droplet aerosol generation for droplet spectrum.** Primary aerosol droplets were generated by usage of the device PARI LC PLUS® (Pari GmbH, Starnberg, Germany). Air was supplied by a PARI BOY® compressor Type 053 (Pari GmbH, Starnberg, Germany), so the liquid from an 8 ml reservoir exits the nozzle producing an aerosol. The size of the released primary droplets depends, among others, on the shear forces inside the nozzle and the salt content of the liquid. Here, results for the use of Ringer's solution (B.Braun, Melsungen, Germany) as dispensing liquid are presented.

**Measurement of droplet spectrum.** Measurements of the droplet spectrum were performed on a closed loop air channel for filtration tests according to DIN EN 779. The air circulating within the channel can be conditioned with regard to humidity and temperature. Conditions during the experiment were 18 °C, 994 Pa and 90% RH inside the duct. Gas velocity in the measurement area was approximetly 0.7 m s^-1^. The droplets were released within the flow, the nozzle orifice of the measurement device (Aerosol spectrometer welas digital 2000H with aerosol sensor welas® 2070 HP, Palas GmbH, Karlsruhe, Germany) was placed in opposite direction of the flow in 16 cm distance, see Fig. 1. The air flow rate of the PARI LC PLUS® generated by the compressor was 3.2 L min^-1^. The selected diameter of the nozzle orifice and the orientation of the measuring probe ensured isokinetic and isoaxial sampling. The particle size measuring range was 0.6 to 40 microns, duration for each measurement run was 120 seconds.

**Production of Cryo stocks.** A strain of *L. pneumophila* Sg 1 Subtype Bellingham (DSMZ 25214) was cultured in liquid BYE growth medium for 1 day at 37 °C. Growth Medium contained 5 g Bacto Yeast Extract (BD, Franklin Lakes, USA), 9 mL Legionella BCYE Supplement (VWR, Radnor, USA) and 500 mL ultrapure water. 0.1 mL of culture were plated on a BYCE plate (Xebios Diagnostics, Düsseldorf, Germany). After 3-4 days three colonies were picked and put in 15 mL liquid BYE growth medium. Afterwards, the bacteria were incubated on a shaker at 37°C overnight. On the next day, the concentrations of the grown bacteria cells were measured. Therefore, total cell count (TCC), ILC and TLC were measured on the rqmicro.Count. To continue, the contamination should not be higher than 10%, which means (TLC/TCC) × 100% ≥ 90%. The desired amount of bacteria cells was put in 100 mL sterile Evian water and was conditioned on a shaker at 25 °C for 48h. After two days the bacteria concentration was measured as described before. The cells in Evian water were 1:1 mixed with the cryobuffer. 1100 µL were pipetted in 2 mL Eppendorf tubes and tubes were stored at -80°C until use.

RESULTS

**Droplet distribution Pari LC Plus.** As result of the aerosol measurements, Figure S1 shows the cumulative mass distribution. 50% of the mass fraction fall within a droplet size range above and below a Mass Mean Diameter (MMD) of 6.3 µm, respectively, whereas 80% of the mass falls in the range between 2.3 to 12 µm. The distribution shows a bimodal character, with modes at 1 µm and 8 µm.

According to a publication by the manufacturer [38], the characteristic Mass Median Aerodynamic Diameter (MMAD) of the PARI LC device family is on an average MMAD value of 4.0 µm to 5.5 µm depending on the air volume flow (measurements by use of sodium fluoride (NaF)- solution 2,5% and by cascade impaction according to DIN EN 13544-1). The cumulative distribution also indicates a bimodal distribution.

The results need to be evaluated in relation to the procedure for the respective measurement. Different standardized procedures or measuring devices may lead to different results and may use different key figures.

**Figure S1.** Cumulative mass distribution for aerosol of Ringer’s solution released by PARI LC PLUS® with a PARI BOY® compressor (air flow rate 3,2 L min^-1^).

**Linear regressions.**

**Table S1. Linear regressions and respective correlation coefficients by IMS-FCM.**

|  | Linear regression (TLC) | ρ (TLC) |
| --- | --- | --- |
| Before aerosolization | y = 1.089x - 0.662 | 0.996 |
| After aerosolization | y = 1.018x - 0.569 | 0.997 |
|  | Linear regression (ILC) | ρ (ILC) |
| Before aerosolization | y = 0.970x - 0.152 | 0.999 |
| After aerosolization | y = 0.957x - 0.310 | 0.996 |

**Table S2.** **Linear regressions and respective correlation coefficients by qPCR.**

|  | Linear regression (TLC) | ρ (TLC) |
| --- | --- | --- |
| Before aerosolization |  |  |
| *L. pneumophila* | y = - 3.086x + 38.898 | -0.997 |
| *L. pneumophila* Sg 1 | y = - 3.177x + 42.282 | -0.997 |
| *Legionella spp.* | y = - 3.270x + 41.310 | -0.997 |
| After aerosolization |  |  |
| *L. pneumophila* | y = - 3.315x + 44.023 | -0.999 |
| *L. pneumophila* Sg 1 | y = - 3.277x + 46.885 | -0.997 |
| *Legionella spp.* | y = - 3.398x + 46.158 | -0.998 |
|  | Linear regression (ILC) | ρ (ILC) |
| Before aerosolization |  |  |
| *L. pneumophila* | y = - 3.355x + 40.144 | -0.999 |
| *L. pneumophila* Sg 1 | y = - 3.459x + 43.528 | -0.998 |
| *Legionella spp.* | y = - 3.565x + 42.719 | -0.998 |
| After aerosolization |  |  |
| *L. pneumophila* | y = - 3.444x + 44.64 | -0.998 |
| *L. pneumophila* Sg 1 | y = - 3.518x + 48.034 | -0.997 |
| *Legionella spp.* | y = - 3.688x + 47.758 | -0.997 |

**Table S3.** **Linear regressions and respective correlation coefficients by cultivation.**

|  | Linear regression | ρ |
| --- | --- | --- |
| Before aerosolization | y = 1.048x - 0.766 | 0.999 |
| After aerosolization | y = 1.035x – 1.341 | 0.950 |
